# Orange juice intake and lipid profile: a systematic review and meta-analysis of randomised controlled trials

**DOI:** 10.1017/jns.2023.22

**Published:** 2023-03-17

**Authors:** Mohammad Reza Amini, Fatemeh Sheikhhossein, Elham Bazshahi, Hamed Rahimi, Hamid Ghalandari, Ehsan Ghaedi, Moein Askarpour

**Affiliations:** 1 Student Research Committee, Department of Clinical Nutrition and Dietetics, Faculty of Nutrition Sciences and Food Technology National Nutrition and Food Technology Research Institute, Shahid Beheshti University of Medical Sciences, Tehran, Iran; 2 Department of Clinical Nutrition, School of Nutritional Sciences and Dietetics, Tehran University of Medical Sciences (TUMS), Tehran, Iran; 3 Department of Community Nutrition, School of Nutritional Sciences and Dietetics, Tehran University of Medical Sciences (TUMS), Tehran, Iran; 4 Department of Community Nutrition, School of Nutritional Sciences and Dietetics, Shiraz University of Medical Sciences (SUMS), Shiraz, Iran; 5 Student Research Committee, Department of Clinical Nutrition, School of Nutrition and Food Sciences, Shiraz University of Medical Sciences, Shiraz, Iran

**Keywords:** Lipid profile, Meta-analysis, Orange juice, RCT

## Abstract

Dyslipidaemia is a metabolic anomaly which has been related to numerous morbidities. Orange juice (OJ) is a popular flavonoid-rich drink consumed worldwide. Due to the existing controversies regarding its impact on blood lipids, we decided to investigate the impact of OJ supplementation on lipid profile parameters. Major scientific databases (Cochrane library, Scopus, PubMed and Embase) were searched. Pooled effects sizes were reported as weighted mean difference (WMD) and 95 % confidence intervals (CIs). Out of 6334 articles retrieved by the initial search, 9 articles met our inclusion criteria. Overall, supplementation with OJ did not exert any significant effects on blood levels of TG (WMD −1·53 mg/dl, 95 % CI −6·39, 3·32, *P* = 0·536), TC (WMD −5·91 mg/dl, 95 % CI −13·26, 1·43, *P* = 0·114) or HDL-C (WMD 0·61 mg/ dl, 95 % CI −0·61, 1·82, *P* = 0·333). OJ consumption did reduce LDL-C levels significantly (WMD −8·35 mg/dl, 95 % CI −15·43, −1·26, *P* = 0·021). Overall, we showed that the consumption of OJ may not be beneficial in improving serum levels of TG, TC or HDL-C. Contrarily, we showed that daily intake of OJ, especially more than 500 ml/d, might be effective in reducing LDL-C levels. In the light of the existing inconsistencies, we propose that further high-quality interventions be conducted in order to make a solid conclusion.

## Introduction

Dyslipidaemia, a common metabolic anomaly and a major risk factor for atherosclerotic cardiovascular disease (ASCVD), comprises elevated low-density lipoprotein cholesterol (LDL-C), non-high-density lipoprotein cholesterol and triglycerides (TG), all of which independently increase the risk of ASCVD^(1)^. Several lifestyle modifications have been recommended for the management of dyslipidaemia, including dietary interventions and increased physical activity^(2)^. Moreover, it has been postulated that the balanced consumption of fruits and vegetables, as a part of healthy dietary patterns, might lead to favourable alterations in lipid profile^(3)^.On the other hand, the global production and consumption of fruit is subpar^(4)^. However, orange juice (OJ) remains one of the most frequently consumed fruit juices worldwide^(5)^, making it the centre of many investigations with regard to diet-related health issues. Natural OJ contains considerable amounts of vitamin C, folate, potassium, fibre and bioactive flavonoids^(6)^, all of which contribute to a healthy diet. Previous studies have shown that the consumption of OJ significantly decreases levels of total cholesterol (TC) and LDL-C^(7)^. Moreover, OJ supplementation decreased LDL-C and increased high-density lipoprotein cholesterol (HDL-C) in both normolipidaemic and hyperlipidaemic participants of a controlled clinical trial^(8)^. The presumed effect of oranges/OJ on improving the lipid profile have been mainly attributed to its flavonoid content, especially hesperidin^(9)^. In this systematic review and meta-analysis of randomised controlled trials (RCTs), we aimed at summing up the existing evidence with regard to potential impact of OJ supplementation on lipid profile.

## Method

### Search strategy

The present study was performed based on the PRISMA guideline^(10)^. A systematic search was conducted via the Cochrane library, Scopus, PubMed and Embase database from inception up to 29 November 2020. The following keywords were used to find the relevant articles: (‘citrus flavonoid’ [tiab] OR orange [tiab] OR ‘citrus sinensis’ [tiab] OR ‘juice’ [tiab]) AND (‘Triglycerides’ [tiab] OR ‘Cholesterol’ [tiab] OR ‘HDL’ [tiab] OR ‘Cholesterol, LDL’ [tiab]) AND (intervention [tiab] OR RCT [tiab] OR ‘controlled trial’ [tiab] ‘Randomised Controlled Trial’ [tiab]). No time or language-restrictions were applied for selection of articles. To make sure that all related works are considered, we also verified the reference list of the articles.

### Eligibility criteria

Original studies were included if they met the following inclusion criteria: (1) being a randomised controlled trial with either parallel or cross-over design, (2) investigating the impact of OJ *v*. placebo on plasma/serum concentrations of lipids and (3) presentation of sufficient information on lipids concentrations at baseline and at the end of follow-up in each group or providing the net change values. Exclusion criteria were (1) non-randomised trials, (2) lack of a placebo control group in the study design, (3) observational studies with case-control, cross-sectional or cohort design and (4) lack of sufficient information on lipid concentrations at the baseline or during the follow-up (or the net change of these parameters).

### Data extraction

Eligible studies were reviewed and the following data were extracted: first author's name, year of publication, the country in which the study was performed, study design, the number of participants in the intervention and placebo groups, the dosage used, treatment duration, age, gender and body mass index (BMI) of study participants, and plasma concentrations of lipid indices at the baseline and during the follow-up.

### Quality of studies

We assigned low risk (L), some concerns (S) and high risk (H) of bias for each six items presented in Table 1, based on Cochrane Collaboration risk of bias tool^(11)^ and according to the following criteria: (1) randomisation process; (2) deviations from the intended interventions; (3) missing outcome data; (4) measurement of the outcome; (5) selection of the reported result and (6) and overall bias (Table 1).

**Table 1.** Risk of bias for randomised controlled trials, assessed according to the Revised Cochrane risk-of-bias tool for randomised trials (RoB 2).

Publications	Randomization process	Deviations from the intended interventions	Missing outcome data	Measurement of the outcome	Selection of the reported result	Overall Bias
1. Aptekmann (2010)	S	S	L	L	L	S
2. Cesar (2010)	S	S	L	L	L	S
3. Morand (2010)	L	L	L	L	L	L
4. Asgary (2014)	S	S	L	L	L	S
5. Constans (2015)	S	S	L	L	L	S
6. Simpson (2016)	S	S	L	L	L	S
7. Gonçalves (2017)	S	S	L	L	L	S
8. Ribeiro (2017)	S	S	L	L	L	S
9. Ponce (2019)	S	S	L	L	L	S

L, low risk of bias; H, high risk of bias; S, some concerns.

### Statistical analysis

To calculate the effect size of the lipid profiles, the mean change and the standard deviation for both the intervention and placebo groups were extracted. A random-effects model (using DerSimonian–Laird method) was used to compensate for the heterogeneity of studies. Standard deviations (sds) of the mean difference were calculated using the following formula: sd change = square root [(sd_pre-treatment_)^2^ + (sd_post-treatment_)^2^−(2 × *R* × sd_pre-treatment_ × sd_post-treatment_)], assuming a correlation coefficient (*R*) = 0·8. If the outcome measures were reported in median and range (or 95 % confidence interval [CI]), mean and sd values were estimated using the method described by Hozo *et al.*^(12)^. Where standard error of the mean (sem) was only reported, sd was estimated using the following formula: sd = sem × sqrt (*n*), where *n* is the number of subjects. Effect sizes were expressed as weighted mean difference (WMD) and 95 % CI. A subgroup analysis according to the trial duration (≤8 or >8 weeks), OJ dose intervention (≤500 or >500 ml/d) and the participants’ gender (both, male, female) was carried out to identify possible sources of heterogeneity. Sensitivity analysis was carried out by omitting each study one by one and recalculating the pooled assesses. Visual inspection of funnel plots, Begg's rank correlation test and Egger's regression test were conducted to identify possible publication bias^(13)^. Statistical analysis was performed using STATA, version 14 (Stata Corp, College Station). *P*-values less than 0·05 were regarded to be statistically significant.

## Result

### Study selection

**Fig. 1.**
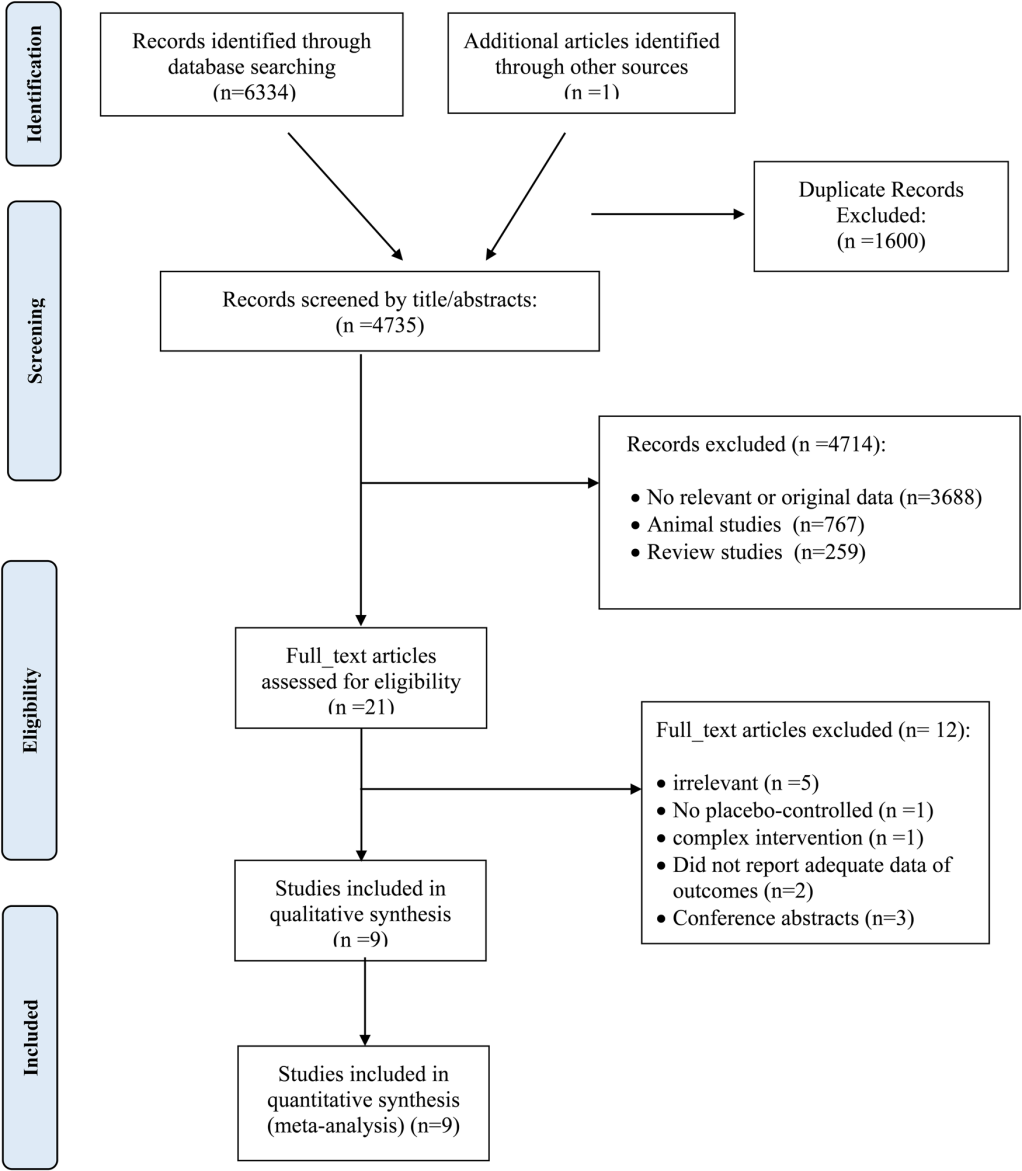
Flow diagram of the study selection.

Initially, after multiple database search, 6334 published studies that were identified (of which 1600 were duplicates) and 4735 reviewed. Of these, 3688 articles did not meet the inclusion criteria and were also excluded. The detailed process of the search strategy is presented in Fig. 1. Finally, nine full RCTs articles, with nine intervention arms were selected for the final meta-analysis.

### Characteristics of studies

Table 2 displays the baseline characteristics of the included studies. In total, 10 effect sizes were extracted from 9 RCTs, including a total of participants of 386. The mean age of the participants ranged from 36 to 56 years. Participants’ health conditions were as follows :healthy^(14)^, patients with hepatitis C^(15)^, hypercholesterolaemic^(16,17)^, normocholesterolaemic^(16)^, overweight^(18–20)^, obese^(21)^ and patients with metabolic syndrome^(22)^. All the RCTs used a parallel study design. The nine eligible articles were released between 2010 and 2019. The RCTs were carried out in Iran^(14)^, France^(17,19)^, UK^(20)^ and Brazil^(15,16,18,21,22)^. The dosage of OJ ranged from 250^(20)^ to 1000 ml/d^(14)^. The duration of interventions ranged from 3^(17)^ to 12 weeks^(18,20–22)^.

**Table 2.** Characteristics of the included studies

First author (year)	Location	Study design	Health status	Gender	Sample size	Duration (week)	Mean age (year)	Baseline BMI (kg/m^2^)	Intervention		Outcome
									Treatment group	Control group	
Cesara (2010) (a)	Brazil	Randomised, placebo-controlled, parallel trial	hypercholesterolaemic	Both	22	8	43·15	26	750 ml orange juice	Placebo	TC/TG/LDL/HDL
Cesara (2010) (b)	Brazil	Randomised, placebo-controlled, parallel trial	Normorcholesterolaemic	Both	39	8	39·9	27·2	750 ml orange juice	Placebo	TC/TG/LDL/HDL
Aptekmann (2010)	Brazil	Randomised, placebo-controlled, parallel trial	Overweight	Female	26	12	39	28·7	500 ml orange juice	Placebo	TC/TG/LDL/HDL
Morand (2010)	France	Randomised, double-blind, placebo-controlled, cross-over	Overweight	Male	24	4	56	27·4	500 ml orange juice	Placebo	TC/TG/LDL/HDL
Asgary (2014)	Iran	Randomised, single-blind, placebo-controlled, cross-over	Healthy	Both	21	4	35·13	24·5	1000 ml orange juice	Placebo	TC/TG/LDL/HDL
Constans (2014)	France	Randomised, single-blind, placebo-controlled, cross-over	Hypercholesterolaemic	Male	25	3	53·8	26	600 ml orange juice	Placebo	TC/TG/LDL/HDL
Simpson (2016)	UK	Randomised, single-blind, placebo-controlled, parallel trial	Overweight	Male	36	12	48·3	31	250 ml orange juice	Placebo	TC/TG/LDL/HDL
Ribeiro (2016)	Brazil	Randomised, non-blind, placebo-controlled, parallel trial	Obese	Both	78	12	36	33	500 ml orange juice and reduced-calorie diet	Reduced-calorie diet	TC/TG/LDL/HDL
Gonçalves (2017)	Brazil	Randomised, placebo-controlled, parallel trial	Patients with hepatitis C	Both	43	8	18<	23·9	500 ml orange juice	Placebo	TC/TG/LDL/HDL
Ponce (2019)	Brazil	Randomised, non-blind, placebo-controlled, parallel trial	MetS	Both	72	12	48	34·6	500 ml orange juice and nutritional guidance	Placebo and nutritional guidance	TC/TG/LDL/HDL

TC, total cholesterol; LDL, low-density lipoprotein; HDL, high-density lipoprotein; TG, triglycerides; MetS, metabolic syndrome.

## Meta-analysis of data

### Effects of OJ on TG

**Fig. 2.**
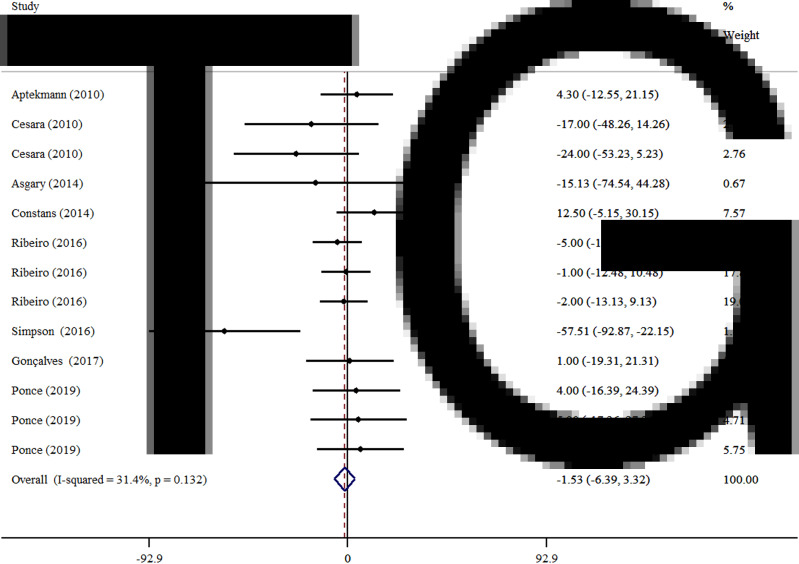
Forest plot for the effect of orange juice on TG concentrations, expressed as mean differences between intervention and control groups. Horizontal lines represent 95 % CIs. Diamond represents the pooled effect size.

As illustrated in Fig. 2, pooling by the fixed-effects model, we could not observe any significant improvement in TG levels (WMD −1·53 mg/dl, 95 % CI −6·39, 3·32, *P* = 0·536) in comparison with the control group. Subgroup analysis was not done for TG, due to not meaningful heterogeneity between studies (*I*^2^ = 45·8 %, *P* = 0·055).

### Effects of OJ on TC

**Fig. 3.**
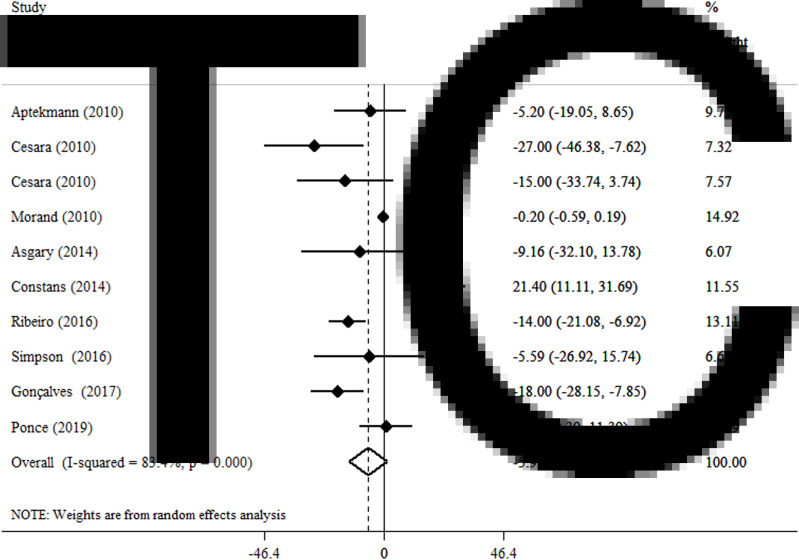
Forest plot for the effect of orange juice on TC concentrations, expressed as mean differences between intervention and control groups. Horizontal lines represent 95 % CIs. Diamond represents the pooled effect size.

By a random effect model, OJ did not significantly reduce TC levels (WMD −5·91 mg/dl, 95 % CI −13·26, 1·43, *P* = 0·114) (Fig. 3). A high level of heterogeneity was observed between the included articles (*I*^2^ = 83·4 %, *P* < 0·001). Subgroup analysis showed that the duration of intervention and gender could justify the high heterogeneity between studies (Table 3).

**Table 3.** Subgroup analysis of included randomised controlled trials in meta-analysis of the effect of orange juice on lipid profile

Group	No. of trials	WMD (95 % CI)	*P*-value^a^	*I*^2^ (%)^b^	*P*-heterogeneity^c^	*P* for between subgroup heterogeneity^d^
TC						
Dosage (ml/d)						0·259
≤500	6	−6·92 (−14·55, 0·70)	0·075	81·5	<0·001	
>500	3	−6·61 (−31·78, 18·57)	0·607	88·5	<0·001	
Duration (week)						0·002
≤8	2	−6·40 (−18·29, 5·49)	0·291	87·2	<0·001	
>8	4	−6·83 (−15·03, 1·37)	0·102	48·4	0·121	
Gender						<0·001
Both	5	−12·75 (−20·19, −5·31)	0·001	50·4	0·073	
Male	3	6·09 (−10·22, 22·41)	0·464	88·3	<0·001	
Female	1	−5·20 (−19·05, 8·65)	0·462	–	–	
LDL						
Dosage (ml/d)						0·012
≤500	6	−7·44 (−16·82, 1·93)	0·120	89·9	<0·001	
>500	3	−9·85 (−18·18, −1·52)	0·020	19·4	0·293	
Duration (week)						<0·001
≤8	5	−7·91 (−15·91, −0·36)	0·040	72·0	0·003	
>8	4	−7·99 (−22·43, 6·44)	0·278	86·0	<0·001	
Gender						<0·001
Both	5	−12·61 (−21·19, −4·04)	0·004	68·9	0·007	
Male	3	−0·77 (−6·18, 4·63)	0·779	38·1	0·199	
Female	1	−10·30 (−25·40, 4·80)	0·181	–	–	
HDL						
Dosage (ml/d)						0·232
≤500	6	0·47 (−0·91, 1·84)	0·506	86·5	<0·001	
>500	3	1·56 (−1·07, 4·19)	0·245	0·0	0·635	
Duration (week)						0·300
≤8	5	−0·39 (−2·63, 1·85)	0·733	56·8	0·041	
>8	4	3·22 (−0·61, 7·05)	0·099	89·1	<0·001	
Gender						<0·001
Both	5	0·20 (−2·80, 3·19)	0·898	66·9	0·010	
Male	1	−0·20 (−1·02, 0·61)	0·624	–	–	
Female	2	12·10 (6·37, 17·83)	<0·001	73·1	0·024	

WMD, weighted mean difference; CI, confidence interval; TC, total cholesterol; TG, triglycerides; HDL, high-density lipoprotein; LDL, low-density lipoprotein.

a Refers to the mean (95 % CI).

b Inconsistency, percentage of variation across studies due to heterogeneity.

c Obtained from the *Q*-test.

d Obtained from the fixed-effects model.

### Effects of OJ on LDL-C

**Fig. 4.**
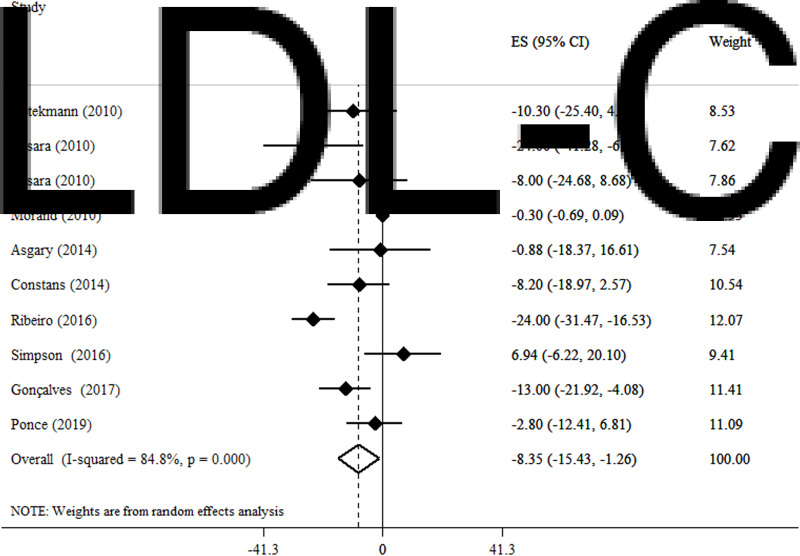
Forest plot for the effect of orange juice on LDL-C, expressed as mean differences between intervention and control groups. Horizontal lines represent 95 % CIs. Diamond represents the pooled effect size.

We found that blood levels of LDL-C (WMD −8·35 mg/dl, 95 % CI −15·43, −1·26, *P* = 0·021) (*I*^2^ = 45·8 %, *P* = 0·055) significantly decrease after OJ supplementation in a random effect model (Fig. 4). In the subgroup analysis based on the administered dosage, LDL-C significantly decreased following the consumption of >500 ml/d OJ (WMD −9·85 mg/dl, 95 % CI −18·18, −1·52, *P* = 0·02). Moreover, the subgroup analyses based on the duration of intervention revealed that the effect of OJ supplementation on LDL-C was significantly greater in trials lasting ≤8 weeks (WMD −7·91 mg/dl, 95 % CI −15·91, −36; *P* = 0·04). Also, studies conducted on both genders were observed to be significantly more likely to reduce blood LDL-C levels (WMD −12·61 mg/dl, 95 % CI −21·19, −4·04; *P* = 0·004) (Table 3).

### Effects of OJ on HDL-C

**Fig. 5.**
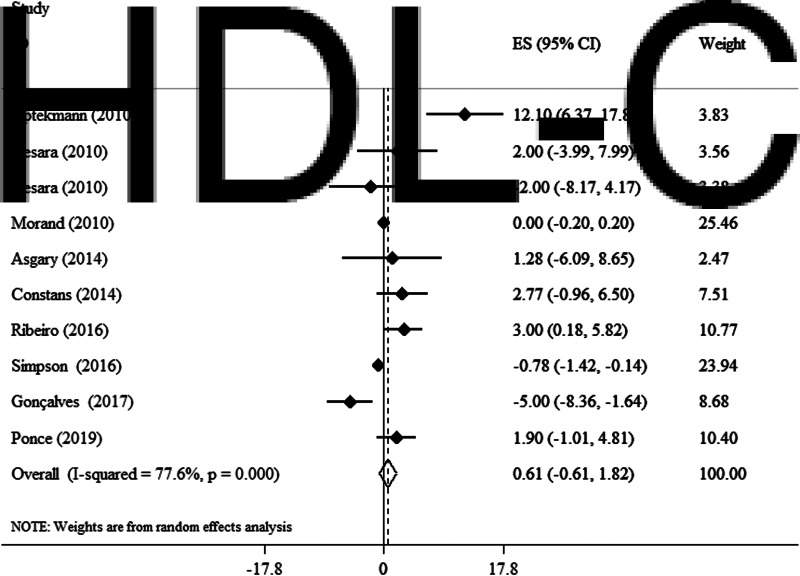
Forest plot for the effect of orange juice on HDL-C concentrations, expressed as mean differences between intervention and control groups. Horizontal lines represent 95 % CIs. Diamond represents the pooled effect size.

In a random effect model, no significant alteration in plasma HDL-C concentrations was observed following OJ supplementation (WMD 0·61 mg/dl, 95 % CI −0·61, 1·82, *P* = 0·333) (*I*^2^ = 77·6 %, *P* < 0·001) (Fig. 5). In subgroup analyses, we observed that OJ significantly reduced HDL-C in studies conducted on women (WMD 12·1 mg/dl, 95 % CI 6·37, 17·83, *P* < 0·001). Further subgroup analyses did not reveal any additional findings (Table 3).

### Publication bias and sensitivity analysis

The sensitivity analysis showed that the estimated overall effect size for lipid profiles did not change dramatically after omitting the size of each effect from the included studies. Based on visual inspection, the funnel plots showed slightly asymmetry for outcomes (Supplementary Figure S1). However, the results of Begg's test suggested no publication bias for TG (*P* = 0·311), TC (*P* = 0·24), LDL-C (*P* = 0·05) or HDL-C (*P* = 0451).

## Discussion

In this systematic review and meta-analysis of 9 RCTs consisting 386 participants which evaluated the effect of OJ on lipid profile, we showed that OJ supplementation significantly reduced blood levels of LDL-C. However, we observed no significant impact for serum levels of TC, HDL-C and TG. Additionally, based on the subgroup analysis, it was found that OJ had a significant effect on LDL-c in doses above 500 ml/d.

Studies have shown that supplementation with OJ leads to significant reductions in the circulating concentration of TC and LDL-C^(23–25)^. Moreover, a large-sized epidemiological study validated the beneficial effects of citrus flavonoids, including hesperidin and naringin, on reducing serum TC concentrations^(7)^. In an experimental model, Kurowska *et al.*^(26)^ showed that in hypercholesterolaemic hamsters, consumption of citrus polymethoxylated flavones (PMFs) (including hesperidin and naringenin) significantly improved lipid profile in both and lower and higher concentrations. In addition, Simpson *et al.*^(20)^ reported no change in LDL levels after supplementation with 250 ml/d of OJ. As stated previously, most of the lipid-lowering effect of oranges is ascribed to their polyphenol content, especially hesperidin^(27)^. On the other hand, in a meta-analysis^(28)^, it was shown that hesperidin intake is not associated with significant changes in blood pressure nor blood lipids. Such conflicting observations may be justified by the difference in the dosage administered or the limited bioavailability of hesperidin. It has been suggested that the remaining of the unabsorbed flavonoid is converted to insoluble compounds by the colon flora^(29)^. One study examined that hesperidin metabolites appeared in plasma 3 h after consuming 440 mg of hesperidin supplement and reached to the peak level after 5–7 h of administration, which can provide 1·28 mmol/l of aglycone hesperidin equivalent^(30)^. Based on that, it is plausible to assume that hesperidin may not reach the sufficient levels that are needed for the regulation of lipid metabolism. Our subgroup analysis confirms these assumptions, since we also showed that OJ consumption had a significant effect on LDL-c only at doses above 500 ml/d.

It is noteworthy that OJ also comprises fair amounts of other flavonoids, such as naringenin and eriodictyol^(31)^. Eriodictyol has been linked to numerous health-promoting impacts, including anti-inflammatory, anti-diabetic and anti-tumor effects^(32)^. However, due to scarcity of high-quality research, its impact on cardiovascular risk factors, including lipid profile, is still unclear. Similarly, naringenin has been claimed to possess anti-inflammatory, anti-adipogenic and cardioprotective effects^(33)^. It is believed that naringenin imposes changes in circulatory lipids, through manipulation of various pathways involved in lipid metabolism; including lipid digestion, reverse transport of cholesterol and reduced reduction of lipoprotein receptors^(34)^. However, in both cases, due to lack of sufficient human trials with respect to their direct effects on measurements of lipid profile, it is difficult to make a general statement regarding their impact on these cardiovascular risk factors.

Lowered blood cholesterol was expected due to the hesperidin and naringin roles as flavonoid components of OJ in reducing the hepatic secretion of very low-density lipoproteins (VLDL) and, consequently, LDL-C into the bloodstream. This LDL-lowering effect of purified citrus flavonoids is supported by *in-vivo* supplementation studies in rodents^(26,35)^, rabbits^(36)^ and humans^(37)^ and the presence of these flavonoids in OJ may contribute to the observation of reduced serum TC levels observed in epidemiological studies^(7)^. These changes lead to increased hepatic receptors that speed up the clearance of circulating LDL-C particles^(24)^. *In-vitro* studies, using isolated liver cells, have shown that citrus flavonoids can reduce net Apo-B secretion, by inhibiting synthesis of the cholesterol esters required for LDL production^(38)^.

As we found in this meta-analysis, OJ consumption had no effect on TG; this observation might be explained by the presence of hesperidin in OJ that has been shown to inhibit pancreatic lipase^(39)^. In a study on subjects with hypercholesterolaemia, increases in plasma TG and HDL-C were produced in response to treatment with 750 ml/d of OJ^(26)^. The authors suggested that the fructose or sucrose content of OJ were not related to this observation, because increases in plasma TG instigated by these sugars in humans were linked with decreases in HDL-cholesterol. In contrast, in another study in which rats were fed a hypercholesterolaemic diet plus a mixture of naringin and hesperidin, no changes in plasma TG levels were reported; indicating that the inhibition of hepatic acyl-CoA cholesterol acyl transferase (ACAT) had no effect on VLDL secretion^(40)^.

Even though the purpose of the present study is not to indicate direct recommendations on the use of 100 % OJ, we find some points worthy of reminding. It is of vital significance how ‘100 %’ or ‘natural’ fruit juices (in this case OJ) are defined. Multiple studies have shown that although 100 % OJ does not seem to augment the risk of CVDs and type 2 diabetes mellitus (T2DM) or even might protect against them^(41,42)^, artificially/sugar-sweetened beverages are considered as potent risk factors and that their health impacts must be distinguished from those of natural fruit juices^(43,44)^. Nonetheless, we find the overall evidence to advocate the daily use of fruit juices (including 100 % OJ) to be lacking and in need of further high-quality investigations.

The present study has some limitations. First, there were considerable between-study heterogeneities. Subgroup analysis, nevertheless, revealed several potential causes of the observed variation including gender, duration of intervention and dosage of OJ. These subgroups analyses are indicators for the fact that, as is the case of all human trials, various interventions with different methods of administration and dosages affect individuals distinctly. Second, the effect of some confounding factors, such as dietary intake and physical activity, were not considered in most of the included studies. Third, most studies were conducted in Brazil, this may exacerbate the potential impacts of other confounding covariates, such as race and eating habits. Third, due to short follow-up period of the included studies, long-term interpretation of the observed effects is limited. Finally, the protocol of the study was not registered in PROSPERO.

## Conclusion

In conclusion, the current systematic review and meta-analysis on available RCTs suggested that OJ consumption might have beneficial effects on blood LDL levels, but no significant effects were observed for serum levels of TC, HDL-C and TG. However, we suggest that further research in the form of high-quality clinical interventions be done, since there are substantial inconsistencies among the included studies.

## Supplementary material

For supplementary material accompanying this paper visit https://doi.org/10.1017/jns.2023.22. click here to view supplementary material
