# Supplementary figures and images for: Orange juice intake and lipid profile: a systematic review and meta-analysis of randomised controlled trials
Source: J Nutr Sci. 2023 Mar 17;12:e37. doi: 10.1017/jns.2023.22 (PMC10052563; doi:10.1017/jns.2023.22)

HDL LDL


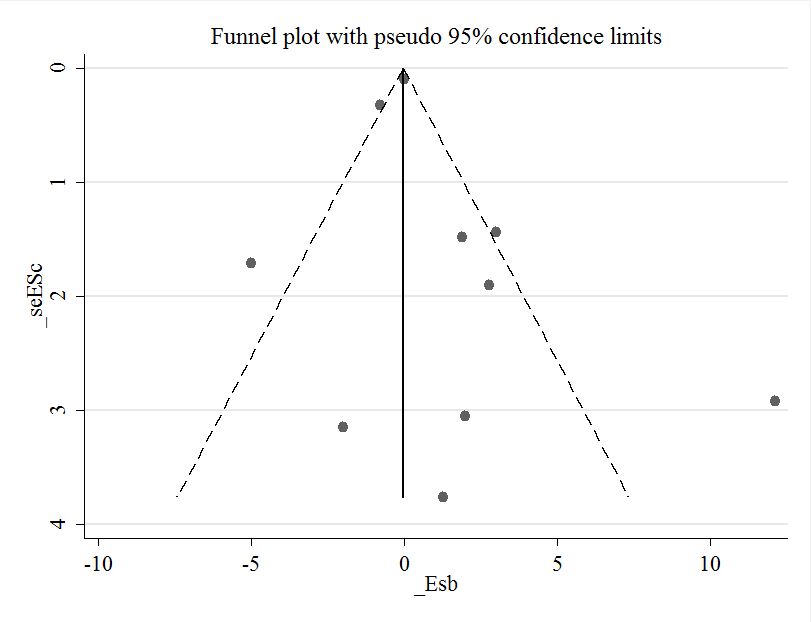

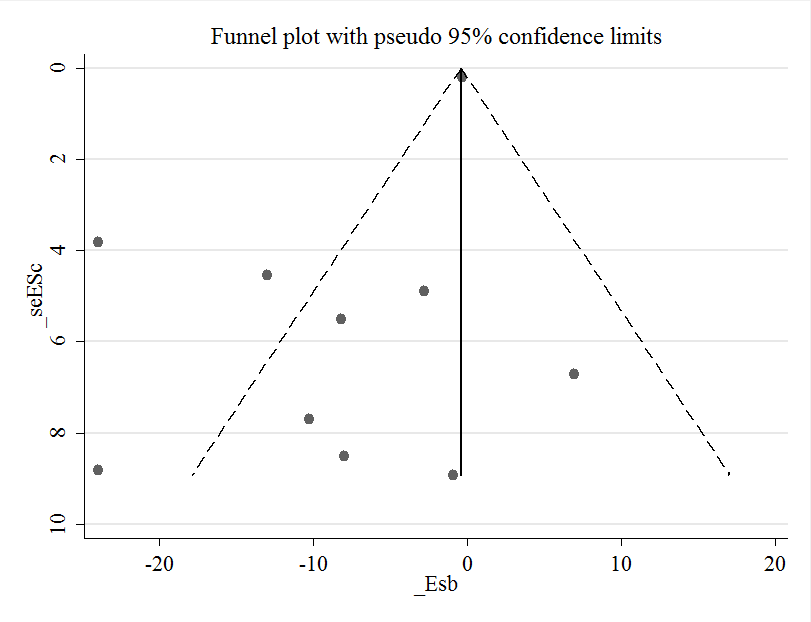


TCTG


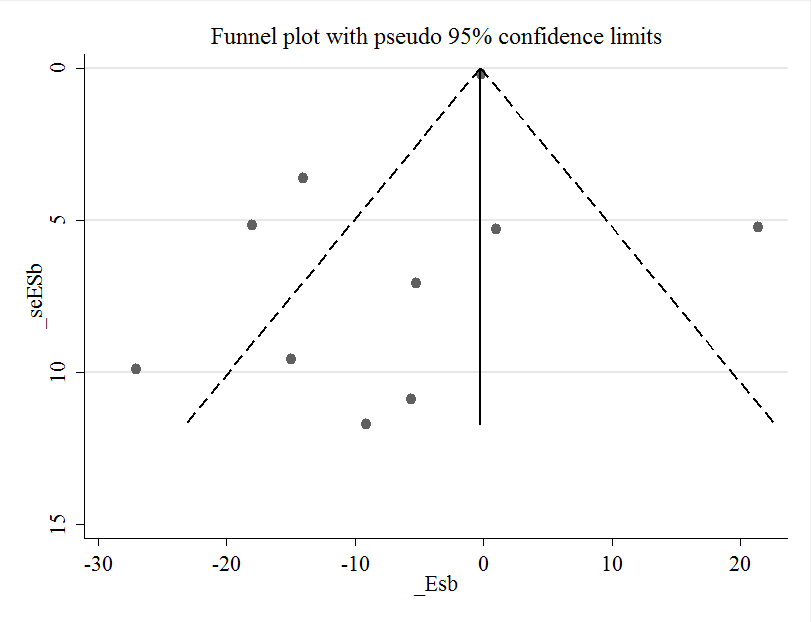

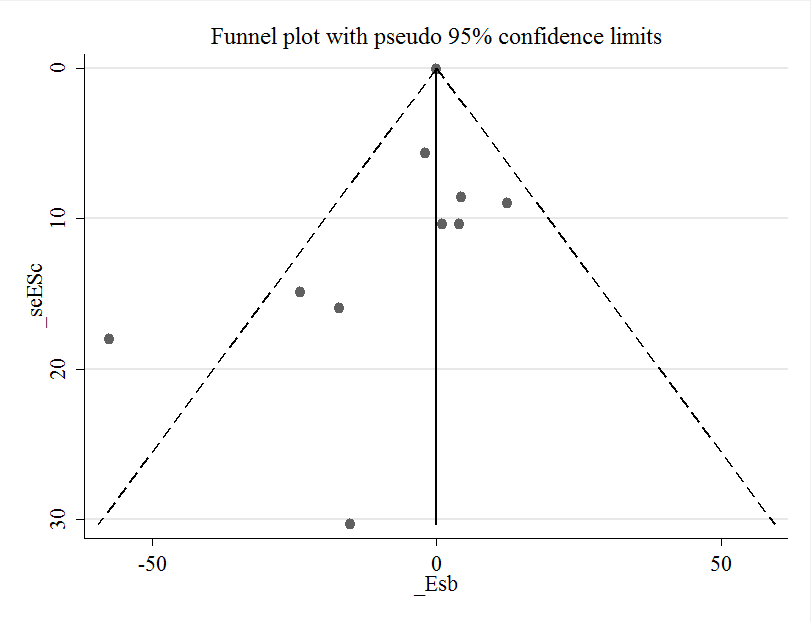

Supplement: Supplementary file 1 [file S2048679023000228sup001.docx]
